# Pharmacological inhibition of the inflammatory receptor CCR2 relieves the early deleterious consequences of status epilepticus

**DOI:** 10.1038/s41598-023-32752-9

**Published:** 2023-04-06

**Authors:** Carlos Alemán-Ruiz, Wenyi Wang, Ray Dingledine, Nicholas H. Varvel

**Affiliations:** 1 Department of Pharmacology and Chemical Biology, Emory University School of Medicine, Atlanta, GA 30322 USA; 2 Present Address: Ponce Health Sciences University, Ponce, PR 00716 USA

**Keywords:** Neuroscience, Cellular neuroscience, Diseases of the nervous system, Neuroimmunology

## Abstract

Generalized status epilepticus (SE) triggers a robust neuroinflammatory response involving reactive astrocytosis, activation of brain-resident microglia, and brain infiltration of CCR2+ monocytes. Multiple lines of evidence indicate that quenching SE-induced neuroinflammation can alleviate the adverse consequences of SE, including neuronal damage and cognitive impairments. Our recent findings show that blocking monocyte brain entry after SE, via global *Ccr2* KO, rescues several SE-induced adverse effects including blood–brain barrier (BBB) erosion, microgliosis and neuronal damage while enhancing weight regain. The goals of the present study were to determine if CCR2 antagonism with a small molecule after SE replicates the effects of the CCR2 knockout. Male *Ccr2*^+*/rfp*^ heterozygous mice were subject to intraperitoneal injection of kainic acid, scored for seizure severity, weight recovery, and nest building capability. Surviving mice were randomized into CCR2 antagonist and vehicle groups. The CCR2 antagonist, or vehicle, was administered 24- and 48-h post-SE via oral gavage, and mice were sacrificed three days post-SE. Mice subject to the CCR2 antagonist displayed faster weight recovery between one- and three-days post-SE and modestly enhanced ability to build a nest on the third day after SE when compared to vehicle-treated controls. CCR2 antagonism limited monocyte recruitment to the hippocampus and reduced numbers of Iba1+ macrophages. The mRNA levels of inflammatory mediators were depressed by 47%, and glial markers were reduced by 30% in mice treated with the CCR2 antagonist compared to controls. Astrocytosis was reduced in four brain regions. Neuroprotection was observed in the hippocampus, and erosion of the BBB was lessened in mice subject to the antagonist. Our findings provide proof-of-concept that brief CCR2 antagonism beginning one day after SE can alleviate multiple adverse SE-induced effects, including functional impairment, and identify circulating CCR2+ monocytes as a viable therapeutic target.

## Introduction

The generalized unremitting seizures of status epilepticus (SE) are a medical emergency that can result in brain injury, death, long-term cognitive deficits, and epilepsy. The available data indicate 41% of survivors of symptomatic SE will develop epilepsy within 10 years^1^. Although information regarding the immediate pathological consequences of acute SE are scarce, hippocampal sclerosis has been observed^2,3^. The inflammatory responses to SE in humans are less well known, but serum albumin was observed in brain of patients who died in SE, indicating erosion of the blood–brain barrier (BBB)^4^. Activation of astrocytes and microglia has been observed in the temporal cortex of a patient with new-onset focal seizures that progressed to refractory SE^5^, and brain-invading monocytes have been observed in hippocampal tissue of epilepsy patients^6^. Elevated CSF levels of chemokines and cytokines, such as IL-6, IL-8, and CXCL10 are typically found in patients with refractory SE compared with patients with other neurologic disorders^7^

Rodent models of SE have provided a wealth of information on the immediate and longer-term pathological and behavioral consequences of SE. SE in rodents provokes transient opening of the BBB, neuronal injury in select brain regions, and robust brain inflammation, typified by activation of brain-resident microglia and astrocytes. Quenching SE-induced neuroinflammation in animal models relieves several deleterious consequences of SE, including neuronal damage, BBB erosion, morbidity, and behavioral deficits^8–12^.

Several inflammatory mediators—cyclooxygenase (Cox-2), IL-1β, and CCL2—are robustly induced within 30 min of SE in mouse hippocampus^13^, and elevated levels of the chemokine, CCL2, have been observed in patients with temporal lobe epilepsy^14^. Induction of the chemokine, CCL2, is notable as it is involved in the recruitment of pro-inflammatory CCR2-expressing monocytes to inflamed tissue in a gradient-dependent manner^15,16^. We and others have demonstrated that germline ablation of *Ccr2* prevents brain infiltration of CCR2-expressing monocytes after SE^17,18^. In addition, SE-induced inflammation was blunted in the global *Ccr2* knockout, microgliosis was inhibited, weight recovery was accelerated, neuronal damage was reduced, and erosion of the BBB was alleviated, supporting the idea that brain invasion by CCR2-expressing monocytes is a pathologic event^17^.

A previous report utilizing a CCR2 antagonist in the rat pilocarpine model of SE showed modest neuroprotection but no change in the rate of behavioral recovery or the number of behaviorally observed spontaneous seizures between 11 and 30 days after pilocarpine SE. Numbers of Iba1+ macrophages appeared unchanged, and monocyte brain entry was not assessed, raising concerns about the effectiveness and mode of action of the CCR2 antagonist treatment regimen^19^.

The aim of the current study was to determine whether pharmacological inhibition of CCR2 with a small molecule antagonist in the days after SE recapitulates the beneficial consequences of global *Ccr2* knockout. Our current findings reveal that CCR2 antagonism after SE effectively blocks monocyte entry into the hippocampus and alleviates the deleterious effects of SE in agreement with our earlier findings employing the *Ccr2* knockout mouse. These findings identify CCR2 as a viable therapeutic target to alleviate the deleterious outcomes of SE and further reinforce the deleterious role of brain-invading monocytes after SE.

## Materials and methods

### Mice

All mice used in the current study were housed in the same rooms in Emory’s animal facility during the course of the experiment, with one room designated for animal breeding and maintenance and an adjacent room solely for SE experiments. The lights were on a 12-h on/off cycle, and mice were fed and watered *ab libitum*. In *Ccr2*^*rfp/rfp*^ KO mice, the *Ccr2* open reading frame was disrupted with a cDNA encoding red fluorescent protein (RFP)^20^. The *Ccr2*^*rfp/rfp*^ mice originally obtained were maintained on the C57BL/6 (Jackson Laboratories) inbred genetic background, and we subsequently backcrossed the mice to C57BL/6 (Charles River) for over 11 generations. To generate heterozygous *Ccr2*^+*/rfp*^ mice, in which Ccr2-expressing monocytes are marked by RFP expression, male *Ccr2*^*rfp/rfp*^ mice were bred to female C57BL/6 mice from Charles River.

After backcrossing for over 11 generations we submitted genetic material obtained from a *Ccr2*^*rfp/rfp*^ mouse to Charles River Laboratories for strain background characterization. A total of 128 single nucleotide polymorphisms (SNPs) spread across the genome was analyzed to generate an allelic profile for comparison between the Jackson Laboratories and Charles River C57BL/6 substrains. Our *Ccr2*^*rfp/rfp*^ mice were a 99.6% match to C57BL/6 inbred mice from Charles River Laboratories, making the mouse strain essentially congenic.

### CCR2 antagonist, INCB3344

INCB3344 is a small molecule antagonist of the chemokine receptor, CCR2, and gifted from Pfizer through their compound sharing program. INCB3344 has favorable selectivity and specificity to CCR2 (target IC_50_ of 10 ± 5 nM; selectivity 300-fold against chemokine receptor CCR5 and CCR1, two close homologues of CCR2, and > 100-fold selective against a broader panel of G protein coupled receptors)^21^. 

### Pharmacokinetics

Pharmacokinetics was performed by Sai Life Sciences Limited (India). Thirty male C57BL/6 mice were divided into two groups (n = 15/group) and administered INCB3344 (30 or 100 mg/kg, p.o.) formulation in 5% NMP, 5% Solutol HS-15, 30% PEG-400, and 60% citric acid (10 mM). Blood samples were collected from retro orbital plexus under light isoflurane anesthesia at 0.5, 1, 2, 4, 8, 12, and 24 h, and brains were collected at 2, 4, 8, 12, and 24 h. Blood was obtained at the 0.5 and 2 h time points from the same three mice, and these mice were sacrificed for brain collection at the 2 h time point. Blood was obtained at the 1 and 4 time points from the same three mice, and these mice were sacrificed for brain collection at the 4 h time point. Blood and brains were collected from three different sets of mice at the 8, 12, and 24 h time points. Plasma samples were separated by centrifugation of whole blood and stored below -70ºC until bioanalysis.

Brain samples were homogenized using ice-cold phosphate buffer saline (pH-7.4) in a ratio of 2 (buffer):1(brain); and homogenates were stored below − 70 ± 10 °C until analysis. All samples were processed for analysis by protein precipitation using acetonitrile (ACN) and analyzed with a fit for purpose LC/MS/MS method (LLOQ: 5.89 ng/mL for plasma and 3.54 ng/g for brain). Pharmacokinetic parameters were calculated using the non-compartmental analysis tool of Phoenix WinNonlin (Version 7.0).

### Kainic acid injection and CCR2 antagonist administration

Kainic acid (KA) was obtained from Tocris and dissolved at 3 mg/ml in a 0.9% sterile saline solution. Heterozygous *Ccr2*^+*/rfp*^ mice were weighed and injected with a single dose of KA (30 mg/kg, i.p.) at 10 ml/kg. In mice, KA-induced seizures consisted of distinct motor behaviors, including forelimb and whole-body clonus, loss of posture, rearing, and falling. Animals that presented these behaviors with increased seizure intensity, duration, and frequency after the injection of KA were declared to be in SE, which is characterized in the KA model by periodic rearing and falling accompanied by whole-body clonic seizures. Behavior was scored using a modified Racine scale as previously described^22^. All mice that entered SE continued seizing for at least 60 min; seizures usually persisted for several hours. The mice were group housed in a warm (28 °C) and humid environment for 12–14 h supplemented with wet food and hydrated with lactated Ringer’s solution i.p when needed, after which they were separated into individual cages.

After 24 h, surviving mice were weighed, randomized into two groups, and received vehicle (5% NMP, 5% Solutol HS-15, 30% PEG-400, and 60% citric acid (10 mM) or the CCR2 antagonist INCB3344 (100 mg/kg, p.o.) at 24 and 48 h after SE onset in a blinded design. The mice were sacrificed three days after SE onset, whole blood was obtained by cardiac puncture prior to perfusion, and the brain was processed for histology, Western analysis, and gene expression as described below.

### Nesting behavior

The ability of a mouse to construct its nest between the second and third day after SE from a supplied nestlet square was assessed on a rating scale of 1–5^23^. 1: the nestlet is more than 90% intact; 2: nestlet is partially torn with 50–90% untouched; 3: more than 50% nestlet is shredded without a nest site; 4: an obvious but flat nest was built; 5: perfect nest with walls higher than mouse body was built.

### Tissue processing

Three days after SE onset, mice were anesthetized deeply with isoflurane, perfused through the heart with PBS solution, and their brains rapidly removed from the cranium. The brain was immediately bisected through the midline, and the left hemisphere was fixed for 24 h in 4% (wt/vol) paraformaldehyde at 4 °C. After 24 h in 4% PFA, the left hemisphere was cryoprotected in 30% (w/v) sucrose in PBS solution. The cryoprotected left hemisphere was then frozen in 2-methylbutane chilled in dry ice and sectioned coronally at 25 μm using a freezing/sliding microtome. The hippocampus and cortex were dissected from the right half of the brain, immediately frozen on dry ice and stored at − 80 °C for RNA isolation and Western blot analysis, respectively.

### Stereological assessment of macrophage and monocyte number

Numbers of Iba1+ macrophages and RFP+ monocytes were assessed on sets of every 12th systematically sampled 25 μm thick coronal immunostained section through the hippocampus. Thus, three sections were analyzed from each mouse. The sections were stained with rabbit polyclonal antibody to Iba1 (1:1000, Wako, 839,504) or RFP (1:1000, Abcam) and Vectastain Elite ABC Kits (Vector Laboratories). Stereological analysis was performed with the aid of the Stereologer software (Stereo Investigator 6, MBF Bioscience) and a motorized x–y–z stage coupled to a video microscopy system. The optical fractionator technique was used with 3D dissectors (area, 350 × 350 μm^2^; height, 8 μm; guard height, 2 μm; counting frame, 50 × 50 μm^2^)^24^. RFP+ monocytes and Iba1+ macrophages with complete soma within the dissector volume were counted.

### Immunohistology

For immunofluorescence staining, free floating sections were blocked for 45 min in 5% goat serum and incubated overnight at 4 °C with antibodies against rabbit anti-Iba1 (1:1000, Wako, 839504) and rat anti-GFAP (1:1000, Invitrogen 143165. Primary antibody incubation was followed by extensive washing with PBS solution followed by incubation with fluorescent AlexaFluor secondary antibodies (1:400, Invitrogen) for 45 min, and washing in PBS solution. Stained brain sections were mounted on slides and mounting medium with DAPI and antifade (Vector H-1200) was applied.

### Astrocytosis

Fluorescent images of the neocortex overlying the hippocampus, amygdala, thalamus, and CA1 and CA3 hippocampus were acquired from Iba-1 and GFAP-stained brain sections with a Carl Zeiss Axio Observer A1 epifluorescence microscope equipped with an AxioCam Mrc5 camera. The camera settings for GFAP-stained sections were -0.09 (brightness), 8.56 (contrast), and 629 ms (exposure time) with the “Moments” threshold setting used in NIH ImageJ Fiji software. The same camera settings and threshold levels were employed for all images stained with a given marker. Percentage area occupied by GFAP staining indicates the area above the threshold level. The percentage area of three sections were averaged to provide a single value for each animal. A mean ± SEM was calculated for each treatment group.

### Western blot analysis of albumin

Cerebral cortices from saline perfused mice were homogenized in 10 volumes of RIPA buffer with protease and phosphatase inhibitors (Thermo Scientific). Brain homogenates were subsequently sonicated to shear DNA then centrifuged at 14,000×*g* for 30 min at 4 °C to remove nuclei and cell debris. Brain protein was run on a 4–20% Mini-PROTEAN TGX Gel and electroblotted onto PVDF membranes (Millipore). Membranes were blocked for one hour at room temperature with Odyssey blocking solution (Li-Cor), then incubated overnight at 4 °C with primary antibodies for albumin (1:1000; Cell Signaling) and GAPDH (1:10,000; Calbiochem), followed by incubation with polyclonal IRDye secondary antibodies 680LT and 800CW (1:15,000; Li-Cor). The blots were imaged by a Li-Cor imaging system using channels 700 and 800. Band intensity was measured and corrected for nearby background intensity by the Li-Cor software. The value of the albumin/GAPDH ratio for each sample was then determined. The average ratio for two groups was compared by t test. The fold-change for each group was referenced to the vehicle-treated group and plotted.

### Fluoro-Jade B labeling

Sections were mounted on Superfrost Plus Microscope Slides (Fisher Scientific) and allowed to air-dry overnight. Sections were immersed in 0.06% potassium permanganate for 15 min with gentle agitation, rinsed for one minute in distilled water, and then transferred to the Fluoro-Jade B staining solution (0.0001% wt/vol FJB in distilled water with 0.1% acetic acid) for 30 min with gentle agitation in the dark. Sections were rinsed with three one-minute changes of distilled water and air-dried. The slides were immersed in xylene and then coverslipped with D.P.X. mounting media (Sigma-Aldrich). Three sections between bregma − 1.34 and − 2.40 were examined with a fluorescent microscope.

Following Fluoro-Jade B staining, images were obtained from three hippocampal areas (hilus, CA1, CA3) in each section. A researcher blinded to treatment and experimental conditions counted the number of Fluoro-Jade B-positive neurons in the hippocampus. Only positive neurons with a near-complete cell body shape and size were tabulated. Cell counts were expressed as the total number of Fluoro-Jade B-positive cells per section for each region.

### qRT-PCR

Total RNA from mouse hippocampus was isolated by using TRIzol (Invitrogen) with the PureLink RNA Mini Kit (Invitrogen). RNA concentration and purity were measured by A260 value and the A260/A280 ratio, respectively. We typically recovered 5–15 µg RNA from each hippocampus with A260/280 ratio = 2.1–2.2. First-strand cDNA synthesis was performed with 1.0 μg of total RNA, using qScript cDNA SuperMix (#95048, Quantabio) in a reaction volume of 20 μL at 25 °C for five minutes, then 42 °C for 30 min. The reaction was terminated by heating at 85 °C for five minutes. qRT-PCR was performed by using eight μL of 10x- or 50x-diluted cDNA, 0.1–0.5 μM of primers, and 2 × iQ SYBR Green Supermix (Bio-Rad Laboratories), with a final volume of 20 μL, in the iQ5 Multicolor Real-Time PCR Detection System (Bio-Rad). Cycling conditions were as follows: 95 °C for two minutes followed by 40 cycles of 95 °C for 15 s and then 60 °C for one minute. Melting curve analysis was used to verify single-species PCR product. Fluorescent data were acquired at the 60 °C step. The geometric mean of the cycle thresholds for *β*-actin, GAPDH, and HPRT1 was used as internal RNA-level control for relative quantification (Table 1). Samples without cDNA served as the negative controls.

**Table 1:** CT values and geometric means of housekeeping genes from mice three days after SE.

Genes	KA vehicle	KA antagonist
HPRT1	29.8 ± 0.3	29.3 ± 0.3
β-actin	28.9 ± 0.5	28.7 ± 0.2
GAPDH	21.9 ± 0.3	21.7 ± 0.1
Geomean	26.6 ± 0.3	25.8 ± 0.2

Data for are the mean ± SEM. (n=20 KA vehicle, n=19 KA antagonist).

### Blood cell analysis

Whole blood was analyzed automatically by using the animal blood counter VetScan HM5 (ABAXIS).

### Experimental design and statistical analysis

The primary objective of the present study was to examine the consequences of systemic CCR2 antagonism after SE utilizing a small chemical molecule. Therefore, the effects of SE in mice subject to the CCR2 antagonist were compared to the effects of SE in vehicle-treated, aged-matched littermates. Prospective power analysis was based on preliminary data showing that CCR2 antagonism blocks monocyte brain entry to a similar degree encountered in *Ccr2* KO mice after SE^17^, and the *Ccr2* KO mice showed a 49% reduction in neuronal damage compared to CCR2-sufficient littermates post-SE resulting in an alpha < 0.05 and power = 0.69. Given these previous findings, the number of mice to achieve a desired power of 0.9 with similar attenuation of neuronal damage would be 19 mice/group. Twenty-four hours after SE, surviving mice were randomized and given CCR2 antagonist or vehicle in a blinded fashion. All assays were performed in a blinded fashion with the experimenter unaware of the treatment group. Just prior to performing statistical tests, potential outliers were identified by Grubb’s test for removal. Unpaired t-tests were used for comparing two groups in Figs. 2C, 3B, 5B, and 8D; an outlier was removed from 3B. Paired t-tests were used for Figs. 7A,B, and 8B. An outlier was removed from 7A and another from 7B. The Mann–Whitney test was performed for evaluating nesting performance in Fig. 2F. The Kruskal–Wallis test followed by Dunn’s correction for multiple comparisons was used in Fig. 6C after removing one statistical outlier from five of the six groups. All outliers, if retained, would not have changed the interpretation. The Kruskal–Wallis test followed by Dunn’s correction for multiple comparisons was used in Figs. 6D. One-way ANOVA followed by Sidak was used in Figs. 3C, 7A,B, and 8E. Two-way ANOVA was used for Fig. 2E.

For analysis of gene induction, the mean ΔΔCt values were compared between selected groups. After statistical analysis, individual ΔΔCt values from each sample group were converted to fold-change by 2^ΔΔCt^, and the fold-change from each group was plotted on a logarithmic scale. Results are expressed as mean values ± SEM. Statistical analysis was performed using GraphPad version 9 (GraphPad Software). For all analyses, the differences were considered to be statistically different if *p* < 0.05.

### Ethical approval

Experimental procedures were performed in accordance with the National Institutes of Health Guide for the Care and Use of Laboratory Animals. The protocol (201900137) was reviewed and approved by the Institutional Animal Care and Use Committee of Emory University. The experiments reported here are in accordance with the ARRIVE guidelines.

## Results

### Pharmacokinetic characterization of CCR2 antagonist

**Figure 1:**
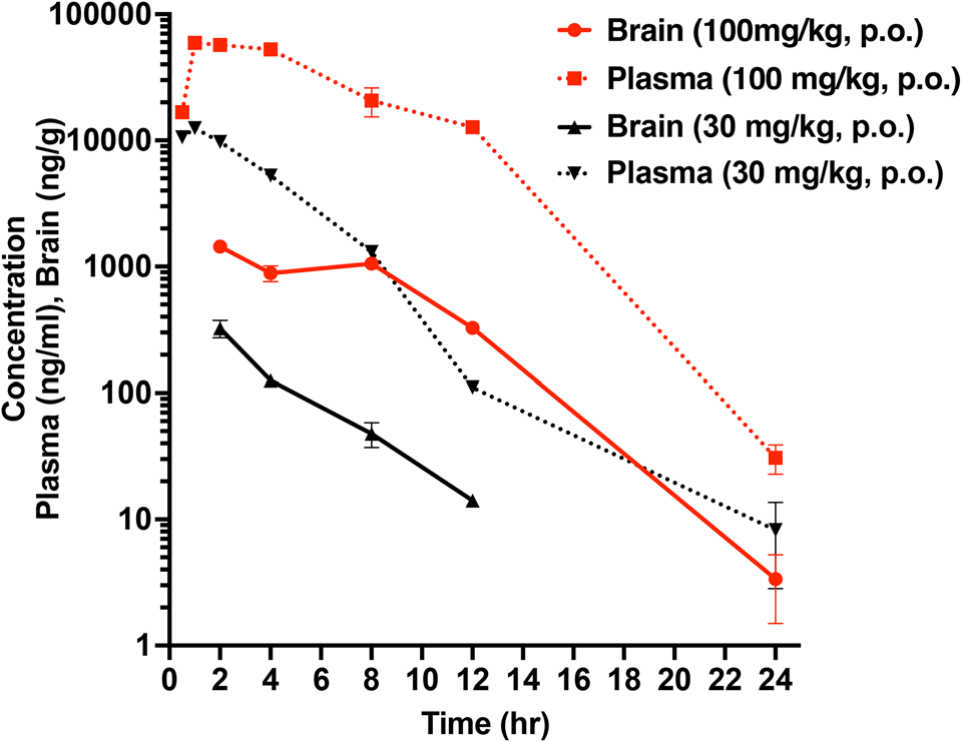
Pharmacokinetic profile of CCR2 antagonist (INCB3344). The CCR2 antagonist was administered to mice (30 or 100 mg/kg, p.o.) for pharmacokinetic studies. The concentrations in the plasma (ng/ml) and brain (ng/g) were measured at time points. Data are shown as mean ± SEM, n = 3 mice per time point.

At the 100 mg/kg dose, the antagonist was detectable in both plasma and brain for 24 h. The CCR2 antagonist displayed a plasma half-life of ~ 1.6 h, the brain half-life was similar to that of plasma, and a brain:plasma ratio 0.02–0.15 (Fig. 1). At the 30 mg/kg dose, the antagonist was quantifiable in plasma for 24 h, but only detected in brain for 12 h. In general, more than dose proportional increase in plasma exposure (4.8-fold for C_max_ and 9.4-fold for AUC_last_) was observed from 30 to 100 mg/kg dose (Table 2).

**Table 2:** Pharmacokinetics of CCR2 antagonist INCB3344 after oral administration.

Dose (mg/kg)	Matrix	T_max_ (hr)	C_max_ (ng/mL)	AUC_last_ (hr*ng/mL)	AUC_inf_ (hr*ng/mL)	T_1/2_ (hr)	CL_f_ (mL/min/kg)	V_Z_ (L/kg)
30	Plasma	1.00	12500	51200	51300	2.60	9.75	2.20
100	Plasma	1.00	59300	481000	481000	1.62	3.46	0.48

Pharmacokinetic profile of CCR2 antagonist INCB3344 in plasma following a single oral dose administration to male C57BL/6 mice (Dose: 30 and 100 mg/kg). CL expressed as CL_f_ and V_ss_ expressed as V_z_.

### CCR2 receptor antagonism accelerates functional recovery after status epilepticus

Given our PK data (Fig. 1 and Table 2) revealing robust bioavailability of the CCR2 antagonist in plasma, and 15% free drug fraction^21^, we calculate that after a single 100 mg/kg dose (p.o.) of the antagonist, the CCR2 receptor will be at least 75% inhibited for 22 h. We investigated the effect of CCR2 receptor inhibition by the antagonist INCB3344 after kainic acid (KA)-induced SE in male heterozygous *Ccr2*^*rfp/*+^ mice maintained on the C57BL/6 inbred background from Charles River.

**Figure 2:**
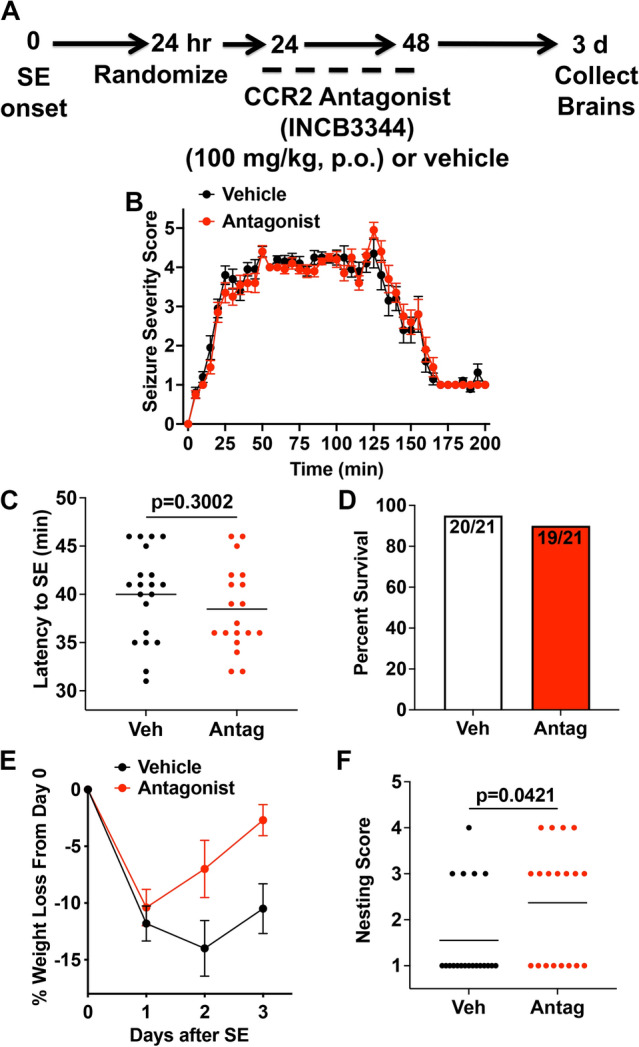
Post-SE treatment with CCR2 antagonist facilitates functional recovery after SE. (**A**) Experimental procedure for post-SE treatment with the CCR2 antagonist. Mice were injected with kainic acid (KA) (30 mg/kg, i.p.) to induce seizures. Twenty-four hours after SE onset the mice were randomized into vehicle (5% NMP, 5% Solutol HS-15, 30% PEG-400, and 60% citric acid (10 mM)) or CCR2 antagonist INCB3344 (100 mg/kg, p.o.). Mice were administered the CCR2 antagonist 24- and 48-h post-SE onset and sacrificed 72 h after SE onset. The mice were checked daily for body weight and mortality. (**B**) The behavioral seizure score was tabulated every 5 min. The temporal evolution of SE was similar in both treatment groups. (**C**) The latency to SE after KA injection, as judged by the behavioral SE score, was similar in vehicle- and CCR2 antagonist-treated mice (the horizontal line is at the mean, symbols represent individual mice; veh n = 20, antag n = 19, unpaired t-test, *p* = 0.300. (**D**) Three-day survival rates of mice that received vehicle or CCR2 antagonist. (**E**) CCR2 antagonist-treated mice (red circles) regained lost body weight faster than vehicle-treated mice (black circles) (veh n = 20, antag n = 19; two-way ANOVA, the effect of treatment, F(1,111) = 10.83, *p* = 0.0013). Data points indicate mean ± SEM. (**F**) Nesting behavior three days after SE (the horizontal line is the mean, symbols represent individual mice; veh n = 20, antag n = 19, Mann–Whitney test, *p* = 0.0421). The achieved power to have detected a real difference was 53%.

Eight batches of eight to 10-week-old *Ccr2*^*rfp/*+^ mice (n = 3–20 per batch) were administered KA. Out of the 63 *Ccr2*^*rfp/*+^ mice that entered SE, 18 mice died in SE. Of the 45 surviving mice, three mice were removed because they did not lose weight 24 h after SE. The remaining 42 surviving mice were randomized to receive either vehicle or CCR2 antagonist (Fig. 2A). The temporal evolution of the seizure scores was similar between vehicle- and antagonist-treated groups (Fig. 2B). Both groups of mice displayed similar latency to enter SE after KA administration. On average, the vehicle-treated mice entered SE 40 min after KA, whereas the antagonist-treated mice entered SE 38.5 min after KA, a difference not statistically significant (Fig. 2C). The similar evolution of seizures and latency to SE between the two groups demonstrates an effective randomization of the two groups prior to administration of the antagonist. Of the 42 surviving mice that lost weight, one mouse died in the vehicle-treated group, and two mice died in the antagonist group (Fig. 2D).

We have previously demonstrated that, during the first 24–48 h following SE, rodents lose 10–20% of their body weight and then slowly begin to recover in the following days^8,11,17,25^. In the current study, the vehicle-treated mice lost 11.8% of their body weight 24 h after SE onset, whereas the antagonist-treated mice lost 10.4% (Fig. 2E). By the second day, antagonist-treated mice began to regain their lost weight and continued to regain weight until they were sacrificed three days after SE. By contrast, vehicle-treated mice continued to lose weight between 24 and 48 h after SE before starting to regain weight (Fig. 2E). There was a statistically significant difference in weight change from day 1 to day 3 between treatment groups (*p* = 0.0013).

Finally, we assessed the ability of the mice to build a nest based on the scoring described in Deacon et al.^23^. CCR2 antagonist-treated mice were slightly better at constructing a nest compared to vehicle-treated mice three days after KA SE. On average antagonist-treated mice scored 2.4 on nesting, whereas control mice received an average score of 1.6 (Fig. 2F). Taken together these findings reveal that the CCR2 antagonist enhances the regain of weight lost by SE and modestly improves nest building capability.

### CCR2 antagonism prevents SE-induced monocyte recruitment to the hippocampus

**Figure 3:**
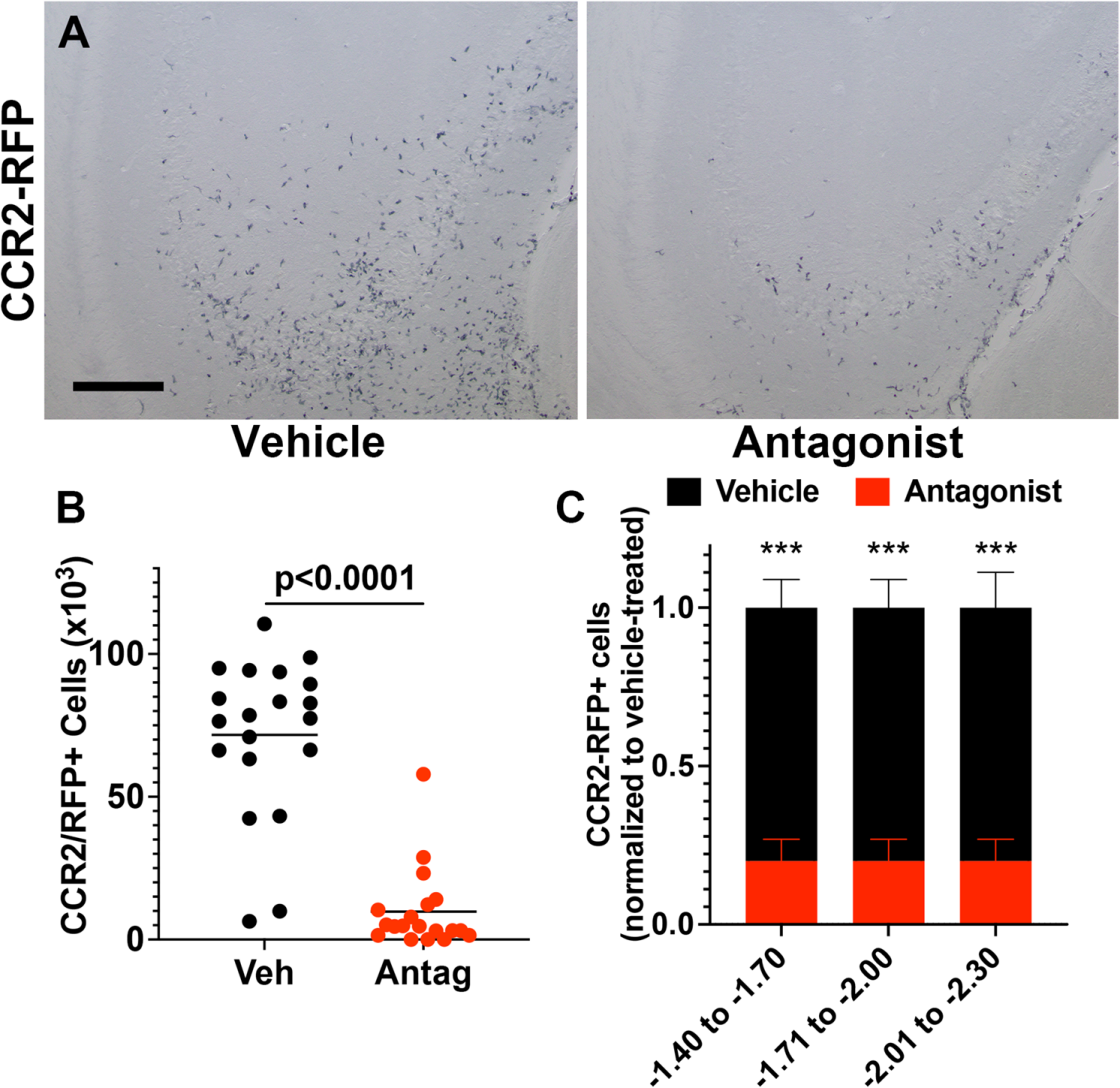
CCR2 antagonist blocks monocyte entry into the hippocampus after SE. (**A**) *Ccr2*^+*/rfp*^ mice (n = 20) subject to KA and vehicle showed hippocampal infiltration of CCR2-RFP + monocytes three days after SE, whereas KA *Ccr2*^+*/rfp*^ mice (n = 18, one statistical outlier removed) subject to the CCR2 antagonist displayed greatly reduced numbers of CCR2-RFP + monocytes in the hippocampus. Scale bar is 200 μm. (**B**) Stereological analysis revealed ~ 71,700 monocytes in vehicle-treated KA mice three days after SE and ~ 9,790 monocytes in CCR2 antagonist-treated KA mice (unpaired t-test, *p* < 0.0001). The horizontal line is at the mean, and each symbol represents data from one mouse. The outlier, if retained, would not have changed the interpretation. (**C**) Plot of the number of CCR2-RFP+ monocytes tabulated and normalized to vehicle-treated controls in the hippocampus segmented across Bregma coordinates listed on the x-axis. One-way ANOVA followed by Holm-Sidak’s comparisons tests found significant differences between treatments in all three regions examined (***p < 0.0001 for each region). Bars indicate mean ± SEM.

Blood-derived monocytes, but not brain-resident microglia, express red fluorescent protein (RFP) in heterozygous *Ccr2*^+*/rfp*^ mice^17,20,26^. Anti-RFP immunostaining and subsequent examination of hippocampal tissues revealed hippocampal invasion of CCR2-RFP-expressing monocytes (Fig. 3A, left). Notably, the CCR2 antagonist greatly reduced the numbers of CCR2-RFP-positive brain-infiltrating monocytes (Fig. 3A, right). Stereological analysis of CCR2-RFP-expressing monocytes showed a mean of ~ 72,000 monocytes in the hippocampus of vehicle-treated KA mice. In contrast, fewer than 10,000 monocytes were tabulated in the SE mice exposed to the CCR2 antagonist (Fig. 3B). Importantly, these data reveal the robust efficacy of the CCR2 antagonist in limiting pathogenic monocyte recruitment to the SE hippocampus, and similar observations were made in the amygdala.

Next, we asked if the observed CCR2 antagonism blocked monocyte brain entry along the rostral-caudal axis of the hippocampus. We grouped our normalized CCR2-RFP monocyte counts from CA1, CA3, and the hilus into three categories corresponding to the tissue section’s Bregma position. CCR2 antagonism reduced the numbers of CCR2-RFP+ monocytes by 80% in each of the regions examined (Fig. 3C).

### Numbers of blood leukocytes are not altered after CCR2 antagonism

**Figure 4:**
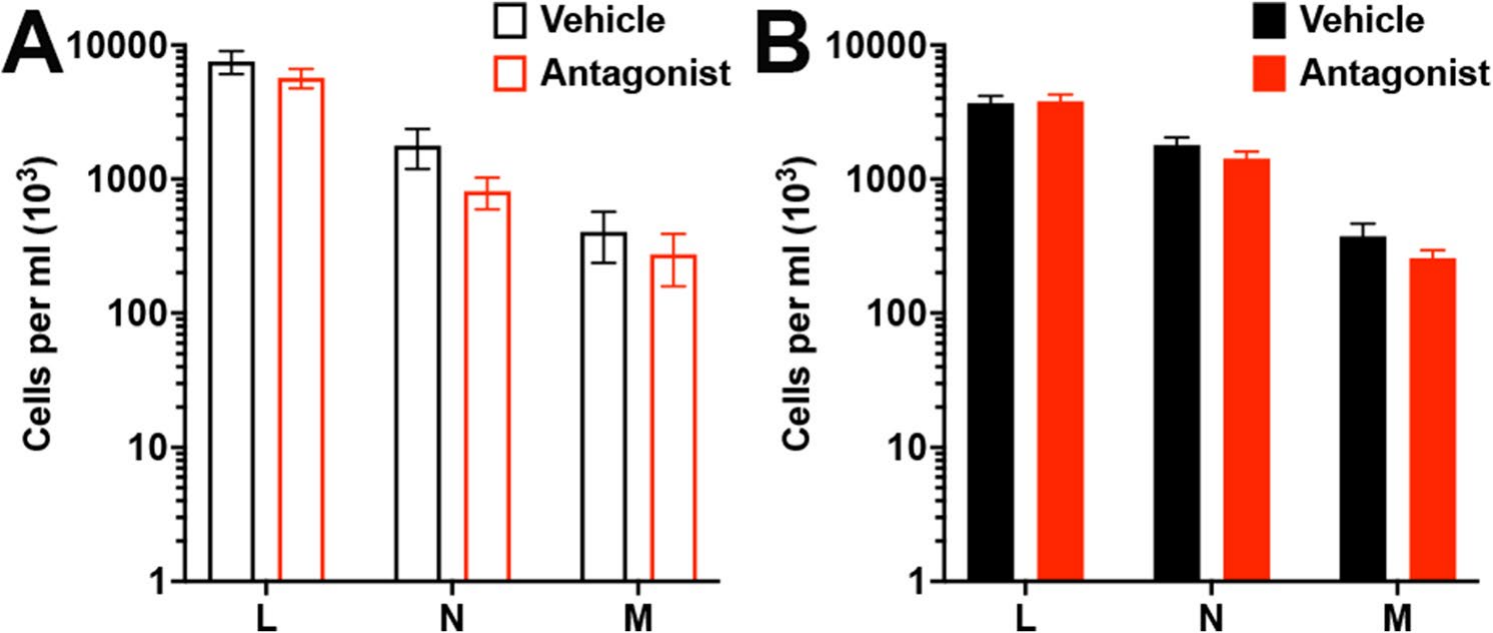
CCR2 antagonism does not change numbers of blood immune cells. (**A**) Blood values of lymphocytes (marked as “L”), neutrophils (“N”), and monocytes (“M”) were not altered between wild-type mice subject to vehicle and the CCR2 antagonist (n = 10/group). (**B**) Blood immune cells from *Ccr2*^+*/rfp*^ mice (n = 20) subject to KA and vehicle were not different from immune cells from KA *Ccr2*^+*/rfp*^ mice (n = 19) subject to the CCR2 antagonist. Bars indicate mean ± SEM.

To examine the possibility that CCR2 antagonism prevents monocyte egress from the bone marrow or in other ways changes the number of immune cells in the blood, we first subjected mice to two doses of the antagonist (or vehicle) 24 h apart and harvested whole blood 24 h after the second dose. Whole blood was analyzed for steady-state numbers of lymphocytes, neutrophils, and monocytes. Notably, administration of the CCR2 antagonist did not change the blood levels of any leukocyte population in naïve, nonSE mice (Fig. 4A) or in mice administered the antagonist after SE (Fig. 4B).

### Serum albumin levels in the cortex are higher after SE in vehicle-treated mice compared to antagonist-treated mice

**Figure 5:**
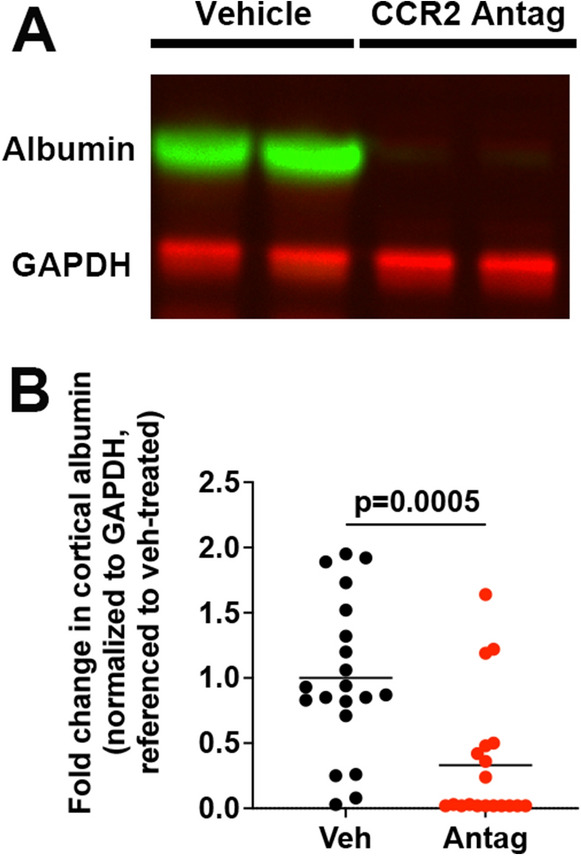
Serum albumin extravasation into the cortex three days after SE was used to evaluate erosion of the BBB. (**A**) Albumin protein levels in cortical homogenates of mice treated with vehicle or CCR2 antagonist after KA-induced SE were measured by Western blot with GAPDH as loading control. Two representative samples from each group are shown. (**B**) Normalized band intensity of the albumin protein relative to that of GAPDH and referenced to the mean albumin/GAPDH ratio in vehicle-treated KA mice. Horizontal bars are at the mean, and each symbol represents data from one mouse. The albumin/GAPDH ratios were compared by unpaired t-test, veh n = 20, antag n = 19. The achieved power to have detected a real difference was 91%.

The BBB is eroded in a number of neurologic conditions including SE, leading to leakage of serum proteins, such as albumin, into the brain. Extravasation of serum albumin into the brain has been reported to initiate the epileptogenic process^27,28^. Global *Ccr2* KO prevents SE-induced albumin entry into the brain^17^. Based on these considerations we examined the effect of the CCR2 antagonist on BBB erosion after SE. Serum albumin was obvious by Western blot analysis in cortex three days after KA-induced SE in vehicle-treated mice, but not in CCR2 antagonist-treated mice (Fig. 5A and Supplemental Figure 1). The average levels of serum albumin detected in cortical homogenates from antagonist-treated mice was 67% lower than mean levels observed in SE mice administered vehicle (Fig. 5B), a statistically significant difference. These findings demonstrate that CCR2 antagonism after SE preserves the integrity of the BBB after SE.

### CCR2 antagonism relieves hippocampal damage after SE

**Figure 6:**
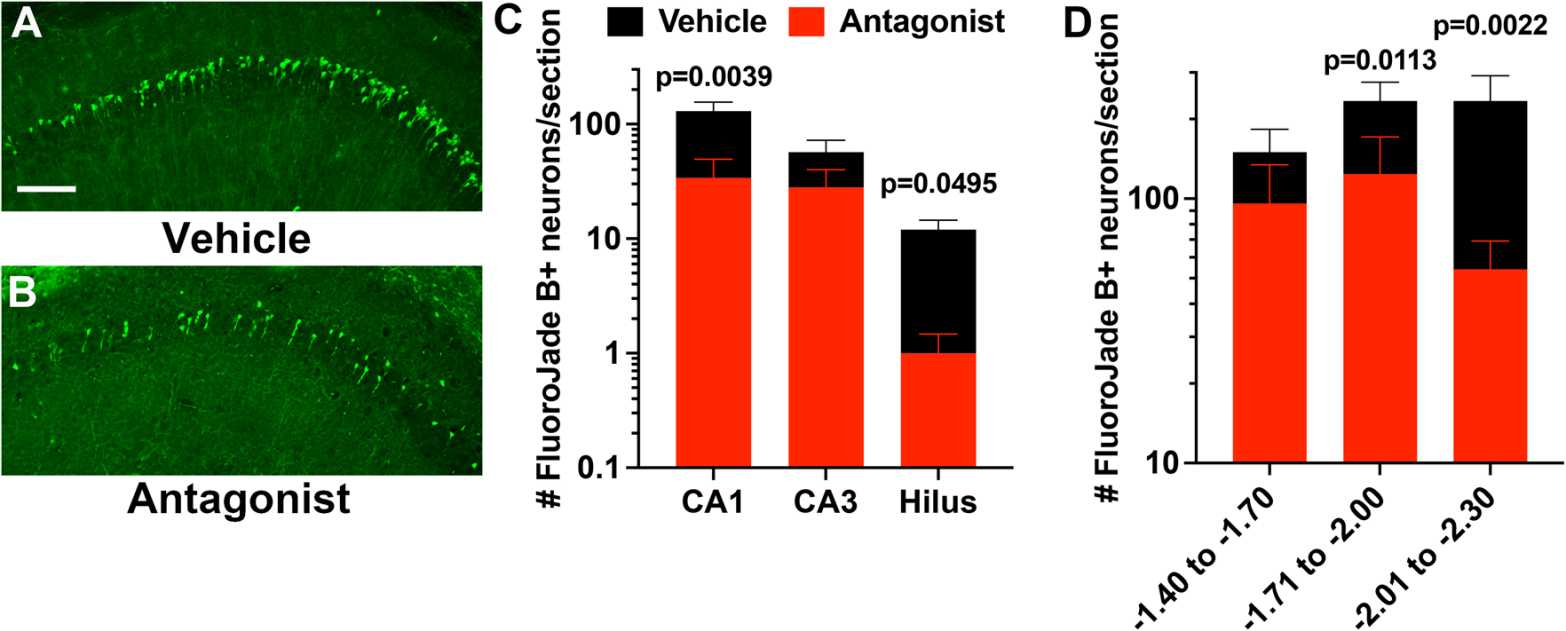
CCR2 antagonism relieves neuronal injury after SE. FJB staining in hippocampal tissue sections three days after KA SE shows reduced numbers of injured CA1 hippocampal neurons in CCR2 antagonist-treated mice (**B**) compared to vehicle-treated mice (**A**). Scale bar is 100 μm. (**C**) Plot of the number of FJB + neurons in hippocampal cell layers reveals fewer damaged neurons in mice subject to the CCR2 antagonist (CA1 veh n = 20; CA1 antag n = 18, one statistical outlier removed; CA3 veh n = 19 statistical outlier removed; CA3 antag n = 18, statistical outlier removed; hilus veh n = 19 statistical outlier removed; hilus antag n = 18, statistical outlier removed). The outliers, if retained, would not have changed the interpretation. Kruskal–Wallis test followed by Dunn’s multiple comparisons tests found significant differences between treatments in the CA1 and hilus, but the reduction in FBJ+ neurons did not reach significance in the CA3 region (*p* = 0.808). Bars indicate mean ± SEM. (**D**) Plot of the total number of FJB+ neurons in the hippocampus segmented across Bregma coordinates. Kruskal–Wallis test followed by Dunn’s multiple comparisons tests found significant differences between treatments in two caudal regions, but the reduction in FBJ+ neurons did not reach significance in the extreme rostral region (*p* = 0.1767). Bars indicate mean ± SEM.

We next evaluated neurodegeneration in hippocampi from mice that received CCR2 antagonist or vehicle 24 and 48 h after SE and were sacrificed three days after SE initiation. Coronal brain sections were stained with Fluoro-Jade B (FJB), and the number of FJB+ neurons in hippocampal subfield cornu ammonis 1 (CA1), CA3, and dentate hilus were determined. KA-induced SE caused substantial hippocampal neuronal injury in CA1, CA3, and hilus in vehicle-treated mice (Fig. 6A). In contrast, FBJ+ neurons were less numerous in antagonist-treated mice (Fig. 6B). This impression was reinforced by counting the number of FJB+ pyramidal neurons in the CA1 and CA3 subfields and hilar neurons. Whereas the numbers of FBJ+ neurons were reduced across all three subfields examined, statistically significant differences were observed in CA1 and hilus (Fig. 6C).

We then asked if the observed neuroprotection was encountered along the rostral-caudal axis of the hippocampus. We grouped our FJB counts from CA1, CA3, and the hilus into three categories corresponding to the tissue sections’ Bregma position. Whereas CCR2 antagonist treatment reduced the numbers of FJB + hippocampal neurons along the entire rostral -caudal axis, neuroprotection was greater in the caudal regions (Fig. 6D). The extreme rostral sections (− 1.40 to − 1.70) did not reach statistical significance. Together, these results support the conclusion that CCR2 receptor activation and consequent monocyte recruitment to the brain promotes neuronal injury after SE, and neuroprotection is observed along the rostral-caudal axis.

### Ccr2 antagonism quenches SE-induced inflammatory burst in the hippocampus

**Figure 7:**
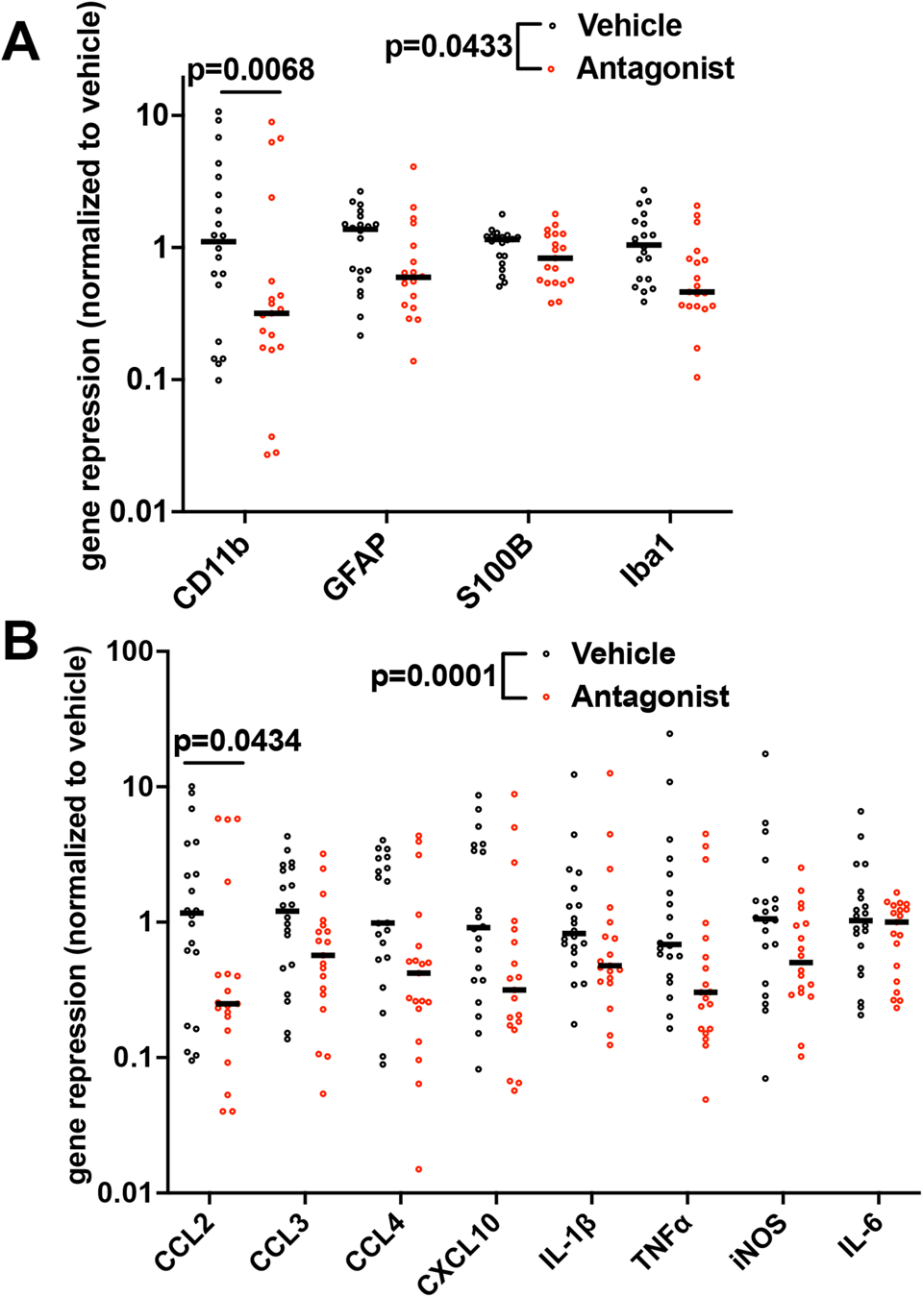
CCR2 antagonism dampens hippocampal glial and inflammatory markers three days after SE. (**A**) Horizontal line indicates median fold repression of the glial mediators in hippocampus and is plotted referenced to the mean of the vehicle-treated group (paired t test, *p* = 0.0433, veh = 20 and antag n = 19 in all groups with one antagonist-treated outlier removed from GFAP group (n = 18)). CD11b was statistically significant (*p* = 0.0068) by one-way ANOVA (the effect of treatment, F(7, 147) = 2.843, *p* = 0.0083) after Sidak correction. The adjusted p value is shown. Symbols represent data from each mouse. (**B**) Horizontal line indicates median fold repression of the inflammatory mediators in hippocampus and is plotted referenced to the mean of the vehicle-treated group (paired t test, *p* = 0.0001, veh = 20 and antag n = 19 in all groups with one antagonist-treated mouse removed from the iNOS group (n = 18)). CCL2 was statistically significant (*p* = 0.0434) by one-way ANOVA (the effect of treatment, F(15, 295) = 2.470, *p* = 0.0020) after Sidak correction. The adjusted p value is shown. Symbols represent data from each mouse.

To ask if CCR2 antagonism broadly dampens the SE-induced glial and inflammatory response, we evaluated the expression profile of a panel of glial (Fig. 7A) and inflammatory genes (Fig. 7B) in hippocampus from mice three days after KA-induced SE. Quantitative real-time PCR (qRT-PCR) was performed on hippocampal tissue to measure the levels of glial markers and inflammatory mediators previously shown to be induced after SE^13,22^. The mRNA levels of astrocytic markers, GFAP and S100B, and microglial/macrophage markers, Iba1 and CD11b, were modestly repressed by 30% in the hippocampus isolated from antagonist-treated mice when compared to vehicle mice (Fig. 7A). The overall repression of the inflammatory genes was 47% in antagonist-treated mice compared to control vehicle-treated mice (Fig. 7B).

Next, we asked which genes are repressed in hippocampus from CCR2 antagonist-treated mice by one-way ANOVA followed by Sidak correction for multiple comparisons. Among the glial markers (ANOVA; the effect of treatment, F(7, 147) = 2.843, p = 0.0083), all four markers were reduced in antagonist-treated mice with CD11b reaching statistical significance (adjusted *p* value, *p* = 0.0068). Among the inflammatory mediators (ANOVA; the effect of treatment, F(15, 295) = 2.470, *p* = 0.0020), seven of eight mediators evaluated were reduced, with CCL2 achieving statistical significance (adjusted *p* value, *p* = 0.0434).

Taken together, these results support the conclusion that CCR2 antagonism broadly dampens glial and inflammatory gene induction in the hippocampus after SE. When evaluated individually and with statistical correction for multiple comparisons, two transcripts, CD11b and CCL2, were found different between treatment groups.

### Astrocytosis and microgliosis are attenuated in CCR2 antagonist-treated SE mice

**Figure 8:**
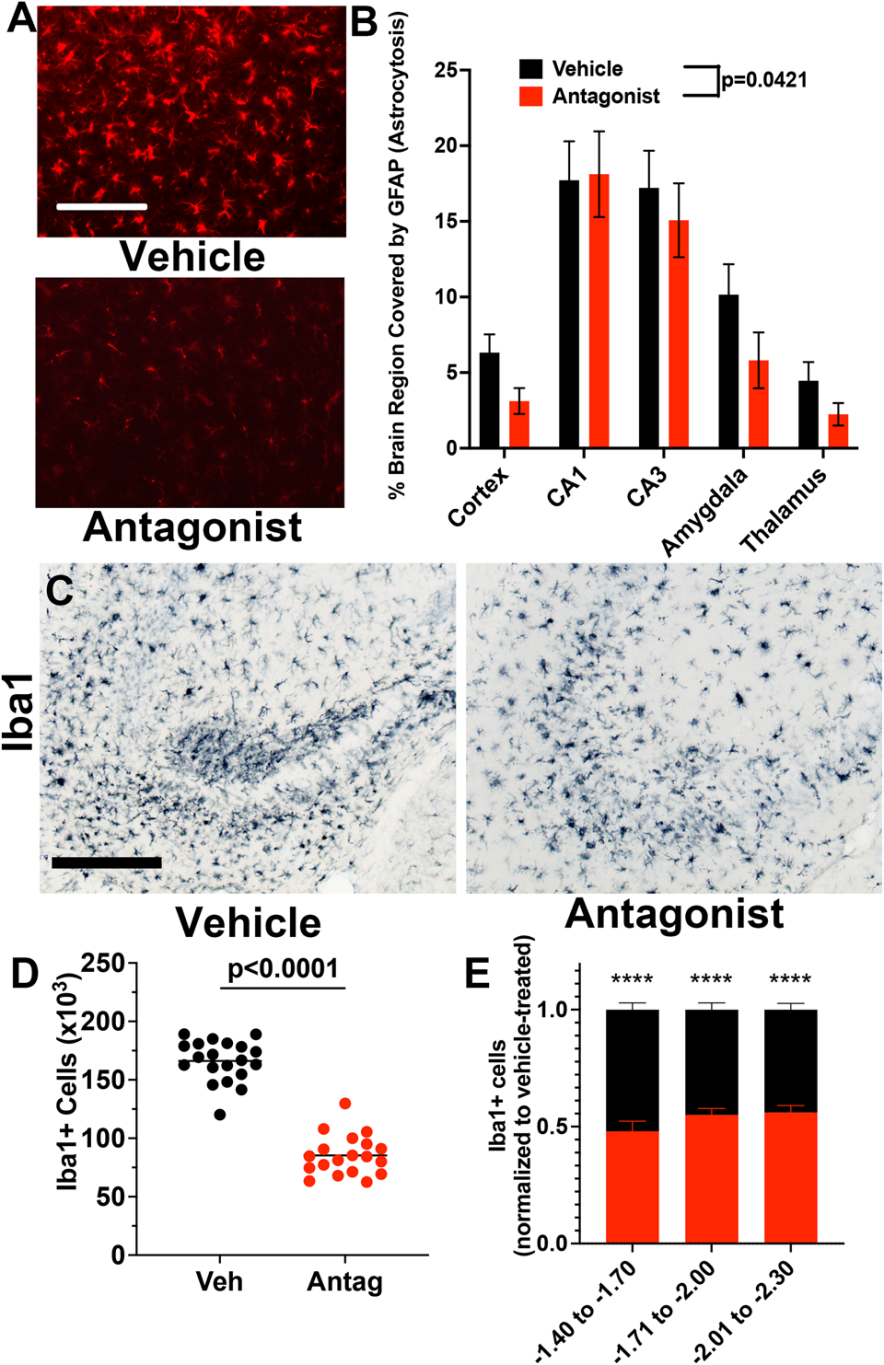
Astrocytosis and microgliosis are dampened in CCR2 antagonist-treated mice. GFAP staining for astrocytes in the amygdala three days after KA SE reveals more gliosis in the vehicle-treated mice (**A**) compared to mice treated with the CCR2 antagonist. Scale bar is 200 μm. Astrocytosis was damped across four out of five brain regions examined in CCR2 antagonist-treated mice (**B**, red bars) when compared to vehicle-treated mice (black bars) n = 18–20/group. Paired t-test, *p* = 0.0421. (**C**) Iba1+ macrophages underwent activation and clustering in both vehicle- and antagonist treated mice. Scale bar is 200 μm. (**D**) Stereological analysis revealed ~ 166,000 Iba1+ cells in the vehicle-treated mice and ~ 85,400 Iba1 + cells in the antagonist-treated mice (unpaired t-test, *p* < 0.0001). Horizontal bars are at the mean, and each symbol represents data from one mouse. (**E**) Plot of the number of Iba1+ macrophages tabulated and normalized to vehicle-treated controls in the hippocampus segmented across Bregma coordinates. One-way ANOVA followed by Holm-Sidak’s comparisons tests found significant differences between treatments in the three regions examined (****p < 0.0001 for each region).

Activation of astrocytes (astrocytosis) is a robust and conspicuous consequence of SE. Coronal brain tissue sections containing neocortex, hippocampus, amygdala, and thalamus were stained with antibody to glial fibrillary acidic protein (GFAP) as a marker of astrocytosis. GFAP staining in the cortex of CCR2 antagonist-treated mice was less intense, and stained cells covered less area compared to vehicle-treated mice (Fig. 8A). Whereas astrocytosis in CCR2 antagonist-treated mice was not changed in CA1 hippocampus, the area covered by GFAP staining was reduced in the cortex, CA3 hippocampus, amygdala, and thalamus. The average reduction in astrocytosis over the five brain regions examined was 31% (Fig. 8B), suggesting that limiting monocyte entry into the brain via CCR2 antagonist mildly reduced astrocyte activation.

Iba1+ macrophages (brain-resident microglia and monocytes) were encountered in both vehicle and CCR2 antagonist mice clustering in the hippocampus (Fig. 8C). However, the numbers of Iba1+ cells in the antagonist-treated mice were reduced compared with the vehicle-treated controls (Fig. 8D). CCR2 antagonism reduced the numbers of Iba1+ cells on average by 52% in the rostral hippocampus, 44% in the caudal hippocampus, and 45% in the central hippocampus (Fig. 8E). Taken together, these findings indicate CCR2 antagonism reduces the numbers of Iba1+ macrophages along the rostral-caudal axis.

Together these results reveal that CCR2 antagonism in the days after kainic acid-induced SE robustly inhibited peripheral monocyte recruitment to the hippocampus, and diminished the early deleterious consequences of SE.

## Discussion

Our study was designed to evaluate the early consequences of pharmacological inhibition of CCR2 after SE in the kainic acid mouse model. We discovered that systemic inhibition of CCR2 beginning 24 h after SE ameliorates the deleterious consequences of SE. CCR2 antagonism prevents SE-induced brain recruitment of peripheral monocytes, enhances weight regain, modestly improves functional recovery as assessed by nesting ability, broadly quenches glial and inflammatory gene induction in the hippocampus, is neuroprotective, and limits deterioration of the BBB after SE. These results demonstrate the participation of pathogenic CCR2+ monocytes in the broad neuroinflammatory response after SE.

The rationale for the current study was pillared on previous findings wherein we and others first reported the involvement of CCR2-expressing monocytes in the neuroinflammatory response in both the kainic acid and pilocarpine SE mouse models^17,18^. Global CCR2-deficient mice subject to pilocarpine SE showed similar seizure intensity as CCR2-sufficient SE mice; however, the *Ccr2* knockout mice had reduced monocyte infiltration into the brain, accelerated weight regain, reduced inflammation and microgliosis in the hippocampus, and less neuronal damage and erosion of the BBB. Strikingly, our pharmacological data with the CCR2 antagonist nearly phenocopies our previous findings with the global CCR2 knockout^17^, confirming the utility of exploiting multiple approaches (i.e., different SE models, genetic and pharmacologic targeting of CCR2) to validate important findings.

A notable difference between our previous findings utilizing the *Ccr2* knockout mouse^17^ and the current data using the CCR2 antagonist to block monocyte brain entry is the extent to which inflammatory transcripts are suppressed in the hippocampus after SE. The hippocampal induction of IL-1β was selectively suppressed among the transcripts analyzed in the *Ccr2* knockout mouse four days after pilocarpine SE. IL-6, TNF, and iNOS were also reduced, but this did not reach statistical significance. CCR2-antagonist treated mice sacrificed three days after SE showed reduced levels in eight of the nine inflammatory mediators analyzed, including IL-1β, with IL-6 showing no change (Fig. 7B).

The reasons for the differences in inflammatory gene suppression are not entirely clear as both the genetic and pharmacologic approaches robustly limited monocyte brain recruitment and reduced the number of Iba1-expressing macrophages. However, it is possible the day the animals were sacrificed (four vs. three days after SE) might be a factor. Another important consideration is the genetic background of the mice as the *Ccr2* knockout mice were on a congenic FVB background in Varvel et al.^17^, whereas the mice subject to the CCR2 antagonist were on a congenic C57BL/6 background. Genetic background can have a profound impact on seizure susceptibility and SE-induced pathologies^29^. Finally, the chemoconvulsants used to induce SE were pilocarpine in Varvel et al.^17^ and kainic acid in the current study. Pilocarpine triggers seizures by muscarinic acetylcholine receptor M1 activation^30^, whereas kainic acid provokes SE by acting on glutamate receptor subtype GluK5 and GluK6^31,32^. Therefore, pilocarpine and kainic acid will elicit differential effects in the central and peripheral compartments. Interestingly, less than 5% of gene expression changes in dentate granule cells observed in three different rat models of SE were in common^33^, indicating fundamental differences among SE models. 

Previous work from our group has documented that systemic antagonism of the prostanoid EP2 receptor or limiting monocyte brain entry, via *Ccr2* KO, alleviates several deleterious SE-induced effects, including a broad and robust burst in glial and inflammatory genes^8,11,13,17,25^. Interestingly, EP2 antagonism after kainic acid-induced SE prevented blood-borne CCR2-expressing monocytes from entering the hippocampus^11^. Notably, in the current study, blocking monocyte brain entry with a CCR2 antagonist resulted in broad suppression of glial and inflammatory gene induction, in similar fashion as previous results with EP2 antagonists^8,13,25^. Moreover, the induction of hippocampal CCL2, a ligand for CCR2 and important for the recruitment of monocytes^16^, is repressed by either EP2 antagonism^8,25^ or CCR2 antagonism (the current study). For these reasons we currently favor the hypothesis that the benefits of EP2 antagonism after SE can be largely attributed to blocking monocyte brain entry.

Our CCR2 antagonist treatments at 24- and 48-h post-SE onset were chosen to ensure robust bioavailability during peak monocyte brain invasion. Notably, whereas inflammatory induction of inflammatory Cox-2, IL-1β, and Ccl2 mRNAs are elevated within 30 min of SE in mouse hippocampus^13^, and activation of the microglial population is evident in tissue sections 24 h post-SE, monocyte recruitment to the brain is delayed, occurring between one and three days post-SE^17^. This delayed monocyte brain recruitment offers a therapeutic window to target monocytes in the days after SE. Nevertheless, the origin of the monocytes remains uncertain. It is possible the monocytes originate from bone marrow or marrow locations in the skull with local access to brain parenchyma^34,35^, but future studies are needed to examine this idea.

Brain invasion of peripheral monocytes has been reported in animal models of other human neurologic disease, such as experimental autoimmune encephalomyelitis (EAE)^36^, traumatic brain injury^37^, ischemic stroke^38^, the Theiler’s murine encephalomyelitis virus (TMEV) model of temporal lobe epilepsy^39^, and Alzheimer’s disease^40,41^. Moreover, elevated numbers of peripheral immune cells have been reported after epileptiform activity^42,43^. Although preventing monocyte invasion after SE was protective (Varvel et al.^17^ and this report), it is possible that CCR2 antagonism might be beneficial in other neurologic diseases accompanied by neuroinflammation and monocyte brain infiltration.

We currently do not know if our antagonist rescues the substantial cognitive comorbidities following KA-induced SE. Our recent work revealed that selectively blocking prostaglandin signaling via systemic antagonism of the EP2 receptor rescued a cognitive co-morbidity in the y-maze 10–14 days after pilocarpine-induced SE^44^, suggesting that selectively quenching inflammation might rescue SE-induced cognitive impairments. It is worth noting that systemic EP2 antagonism robustly blocked monocyte invasion into the brain after SE^11^. Taken together these findings further identify brain-invading monocytes as pathogenic after SE and suggest that brain monocytes might contribute to cognitive co-morbidities following SE.

## Conclusions

Selective pharmacological inhibition of the inflammatory CCR2 chemokine receptor enhances recovery of weight, improves nest building capability, provides neuroprotection, is anti-inflammatory, maintains the integrity of the BBB, and, importantly, prevents the recruitment of CCR2-expressing monocytes to the brain after SE. Taken together these findings reinforce the deleterious role played by brain-infiltrating CCR2-expressing monocytes, and provide evidence supporting the idea that CCR2 antagonism after SE could represent a novel strategy for alleviating the immediate deleterious effects of SE.

## Supplementary Information

Supplementary Figure 1.

## Data Availability

Any data generated and analyzed during this study that are not included in this published article and its additional information will be provided upon reasonable request to the corresponding author.

## Abbreviations

SE: Status epilepticus
BBB: Blood–brain barrier
RFP: Red fluorescent protein
Iba1: Ionized calcium-binding adapter molecule 1
CSF: Cerebrospinal fluid
Cox-2: Cyclooxygenase-2
NMP: *N*-Methylpyrroidone
KA: Kainic acid
PBS: Phosphate buffered saline
GFAP: Glial fibrillary acidic protein
GAPDH: Glyceraldehyde 3-phosphate dehydrogenase
FJB: Fluoro-Jade B
HPRT1: Hypoxanthine–guanine phosphoribosyltransferase
CA1: Cornu ammonis 1
CA3: Cornu ammonis 3
PCR: Polymerase chain reaction
qRT-PCR: Quantitative real-time polymerase chain reaction

## References

[1] Hesdorffer DC, Logroscino G, Cascino G, Annegers JF, Hauser WA (1998). Risk of unprovoked seizure after acute symptomatic seizure: Effect of status epilepticus. Ann. Neurol..

[2] Lewis DV, Shinnar S, Hesdorffer DC, Bagiella E, Bello JA, Chan S (2014). Hippocampal sclerosis after febrile status epilepticus: The FEBSTAT study. Ann. Neurol..

[3] Fujikawa DG, Itabashi HH, Wu A, Shinmei SS (2000). Status epilepticus-induced neuronal loss in humans without systemic complications or epilepsy. Epilepsia.

[4] van Vliet EA, da Costa AS, Redeker S, van Schaik R, Aronica E, Gorter JA (2007). Blood-brain barrier leakage may lead to progression of temporal lobe epilepsy. Brain.

[5] Juhasz C, Buth A, Chugani DC, Kupsky WJ, Chugani HT, Shah AK (2013). Successful surgical treatment of an inflammatory lesion associated with new-onset refractory status epilepticus. Neurosurg. Focus.

[6] Broekaart DWM, Anink JJ, Baayen JC, Idema S, de Vries HE, Aronica E (2018). Activation of the innate immune system is evident throughout epileptogenesis and is associated with blood-brain barrier dysfunction and seizure progression. Epilepsia.

[7] Sakuma H, Tanuma N, Kuki I, Takahashi Y, Shiomi M, Hayashi M (2015). Intrathecal overproduction of proinflammatory cytokines and chemokines in febrile infection-related refractory status epilepticus. J. Neurol. Neurosurg. Psychiatry.

[8] Jiang J, Quan Y, Ganesh T, Pouliot WA, Dudek FE, Dingledine R (2013). Inhibition of the prostaglandin receptor EP2 following status epilepticus reduces delayed mortality and brain inflammation. Proc. Natl. Acad. Sci. U. S. A..

[9] Rojas A, Amaradhi R, Banik A, Jiang C, Abreu-Melon J, Wang S (2021). A novel second-generation EP2 receptor antagonist reduces neuroinflammation and gliosis after status epilepticus in rats. Neurotherapeutics.

[10] Maroso M, Balosso S, Ravizza T, Iori V, Wright CI, French J (2011). Interleukin-1beta biosynthesis inhibition reduces acute seizures and drug resistant chronic epileptic activity in mice. Neurotherapeutics.

[11] Varvel NH, Espinosa-Garcia C, Hunter-Chang S, Chen D, Biegel A, Hsieh A (2021). Peripheral myeloid cell EP2 activation contributes to the deleterious consequences of status epilepticus. J. Neurosci..

[12] Schartz ND, Sommer AL, Colin SA, Mendez LB, Brewster AL (2019). Early treatment with C1 esterase inhibitor improves weight but not memory deficits in a rat model of status epilepticus. Physiol. Behav..

[13] Jiang J, Yang MS, Quan Y, Gueorguieva P, Ganesh T, Dingledine R (2015). Therapeutic window for cyclooxygenase-2 related anti-inflammatory therapy after status epilepticus. Neurobiol. Dis..

[14] Wu Y, Wang X, Mo X, Xi Z, Xiao F, Li J (2008). Expression of monocyte chemoattractant protein-1 in brain tissue of patients with intractable epilepsy. Clin. Neuropathol..

[15] Prinz M, Priller J (2010). Tickets to the brain: Role of CCR2 and CX3CR1 in myeloid cell entry in the CNS. J. Neuroimmunol..

[16] Charo IF, Ransohoff RM (2006). The many roles of chemokines and chemokine receptors in inflammation. N. Engl. J. Med..

[17] Varvel NH, Neher JJ, Bosch A, Wang W, Ransohoff RM, Miller RJ (2016). Infiltrating monocytes promote brain inflammation and exacerbate neuronal damage after status epilepticus. Proc. Natl. Acad. Sci. U. S. A..

[18] Tian DS, Peng J, Murugan M, Feng LJ, Liu JL, Eyo UB (2017). Chemokine CCL2-CCR2 signaling induces neuronal cell death via STAT3 activation and IL-1beta production after status epilepticus. J. Neurosci..

[19] Foresti ML, Arisi GM, Campbell JJ, Mello LE (2020). Treatment with CCR2 antagonist is neuroprotective but does not alter epileptogenesis in the pilocarpine rat model of epilepsy. Epilepsy Behav..

[20] Saederup N, Cardona AE, Croft K, Mizutani M, Cotleur AC, Tsou CL (2010). Selective chemokine receptor usage by central nervous system myeloid cells in CCR2-red fluorescent protein knock-in mice. PLoS ONE.

[21] Brodmerkel CM, Huber R, Covington M, Diamond S, Hall L, Collins R (2005). Discovery and pharmacological characterization of a novel rodent-active CCR2 antagonist, INCB3344. J. Immunol..

[22] Rojas A, Gueorguieva P, Lelutiu N, Quan Y, Shaw R, Dingledine R (2014). The prostaglandin EP1 receptor potentiates kainate receptor activation via a protein kinase C pathway and exacerbates status epilepticus. Neurobiol. Dis..

[23] Deacon RM (2006). Assessing nest building in mice. Nat. Protoc..

[24] Long JM, Kalehua AN, Muth NJ, Calhoun ME, Jucker M, Hengemihle JM (1998). Stereological analysis of astrocyte and microglia in aging mouse hippocampus. Neurobiol. Aging.

[25] Rojas A, Ganesh T, Lelutiu N, Gueorguieva P, Dingledine R (2015). Inhibition of the prostaglandin EP2 receptor is neuroprotective and accelerates functional recovery in a rat model of organophosphorus induced status epilepticus. Neuropharmacology.

[26] Mizutani, M., Pino, P. A., Saederup, N., Charo, I. F., Ransohoff, R. M., Cardona, A. E. The fractalkine receptor but not CCR2 is present on microglia from embryonic development throughout adulthood. *J. Immunol.* (2011). 10.4049/jimmunol.1100421 PMC3244524 22079990

[27] Varvel NH, Jiang J, Dingledine R (2015). Candidate drug targets for prevention or modification of epilepsy. Annu. Rev. Pharmacol. Toxicol..

[28] Ivens S, Kaufer D, Flores LP, Bechmann I, Zumsteg D, Tomkins O (2007). TGF-beta receptor-mediated albumin uptake into astrocytes is involved in neocortical epileptogenesis. Brain.

[29] Schauwecker PE (2011). The relevance of individual genetic background and its role in animal models of epilepsy. Epilepsy Res..

[30] Bymaster FP, Carter PA, Yamada M, Gomeza J, Wess J, Hamilton SE (2003). Role of specific muscarinic receptor subtypes in cholinergic parasympathomimetic responses, in vivo phosphoinositide hydrolysis, and pilocarpine-induced seizure activity. Eur. J. Neurosci..

[31] Ben-Ari Y. Kainate and temporal lobe epilepsies: 3 decades of progress. *Jasper’s Basic Mech. Epilepsies* (2012). 22787646

[32] Mulle C, Sailer A, Perez-Otano I, Dickinson-Anson H, Castillo PE, Bureau I (1998). Altered synaptic physiology and reduced susceptibility to kainate-induced seizures in GluR6-deficient mice. Nature.

[33] Dingledine R, Coulter DA, Fritsch B, Gorter JA, Lelutiu N, McNamara J (2017). Transcriptional profile of hippocampal dentate granule cells in four rat epilepsy models. Sci. Data.

[34] Cugurra A, Mamuladze T, Rustenhoven J, Dykstra T, Beroshvili G, Greenberg ZJ (2021). Skull and vertebral bone marrow are myeloid cell reservoirs for the meninges and CNS parenchyma. Science.

[35] Mazzitelli JA, Smyth LCD, Cross KA, Dykstra T, Sun J, Du S (2022). Cerebrospinal fluid regulates skull bone marrow niches via direct access through dural channels. Nat. Neurosci..

[36] Ajami B, Bennett JL, Krieger C, McNagny KM, Rossi FM (2011). Infiltrating monocytes trigger EAE progression, but do not contribute to the resident microglia pool. Nat. Neurosci..

[37] Gyoneva S, Kim D, Katsumoto A, Kokiko-Cochran ON, Lamb BT, Ransohoff RM (2015). Ccr2 deletion dissociates cavity size and tau pathology after mild traumatic brain injury. J. Neuroinflammation.

[38] ElAli A, Jean LN (2016). The role of monocytes in ischemic stroke pathobiology: New avenues to explore. Front. Aging Neurosci..

[39] Howe CL, LaFrance-Corey RG, Overlee BL, Johnson RK, Clarkson BDS, Goddery EN (2022). Inflammatory monocytes and microglia play independent roles in inflammatory ictogenesis. J. Neuroinflammation.

[40] Mildner A, Schlevogt B, Kierdorf K, Bottcher C, Erny D, Kummer MP (2011). Distinct and non-redundant roles of microglia and myeloid subsets in mouse models of Alzheimer’s disease. J. Neurosci..

[41] Lebson L, Nash K, Kamath S, Herber D, Carty N, Lee DC (2010). Trafficking CD11b-positive blood cells deliver therapeutic genes to the brain of amyloid-depositing transgenic mice. J. Neurosci..

[42] Borges K, McDermott DL, Dingledine R (2004). Reciprocal changes of CD44 and GAP-43 expression in the dentate gyrus inner molecular layer after status epilepticus in mice. Exp. Neurol..

[43] Zattoni M, Mura ML, Deprez F, Schwendener RA, Engelhardt B, Frei K (2011). Brain infiltration of leukocytes contributes to the pathophysiology of temporal lobe epilepsy. J. Neurosci..

[44] Varvel NH, Amaradhi R, Espinosa-Garcia C, Duddy S, Franklin R, Banik A (2023). Preclinical development of an EP2 antagonist for post-seizure cognitive deficits. Neuropharmacology.

