# Supplementary material for: Pharmacological inhibition of the inflammatory receptor CCR2 relieves the early deleterious consequences of status epilepticus
Source: Sci Rep. 2023 Apr 6;13:5651. doi: 10.1038/s41598-023-32752-9 (PMC10079855; doi:10.1038/s41598-023-32752-9)
Supplement: Supplementary file 1 — Supplementary Figure 1. [file 41598_2023_32752_MOESM1_ESM.pdf]

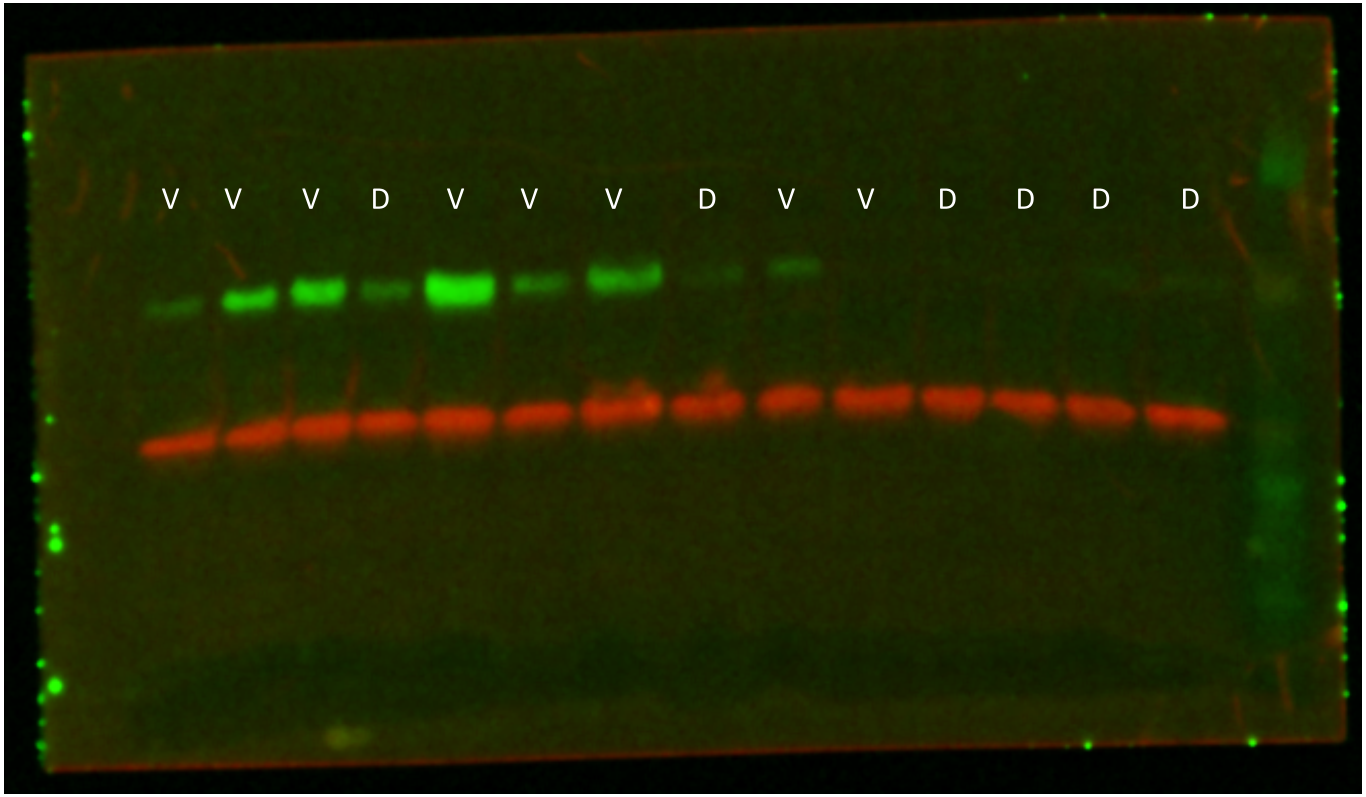
V V V D V V V D V V D D D D

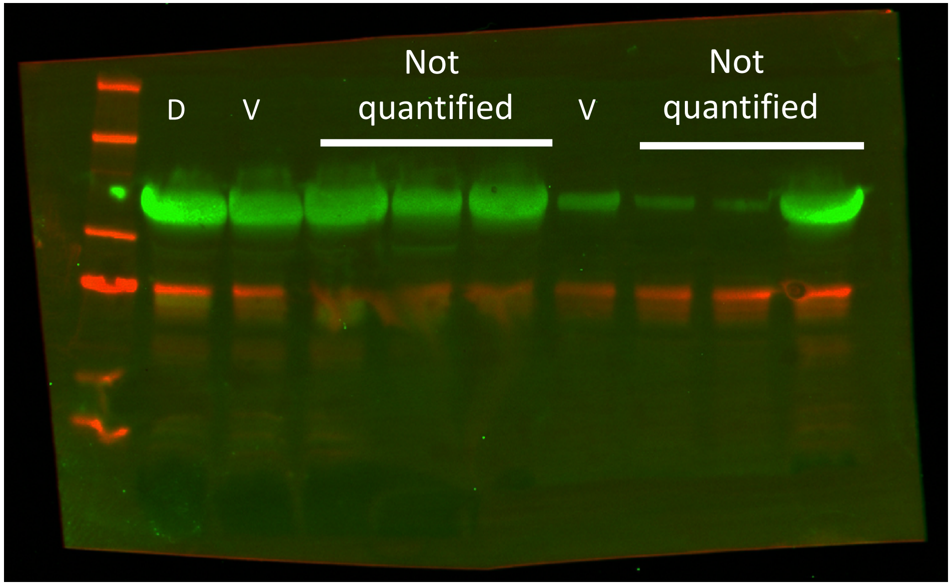
D V
Not quantified
V
Not quantified

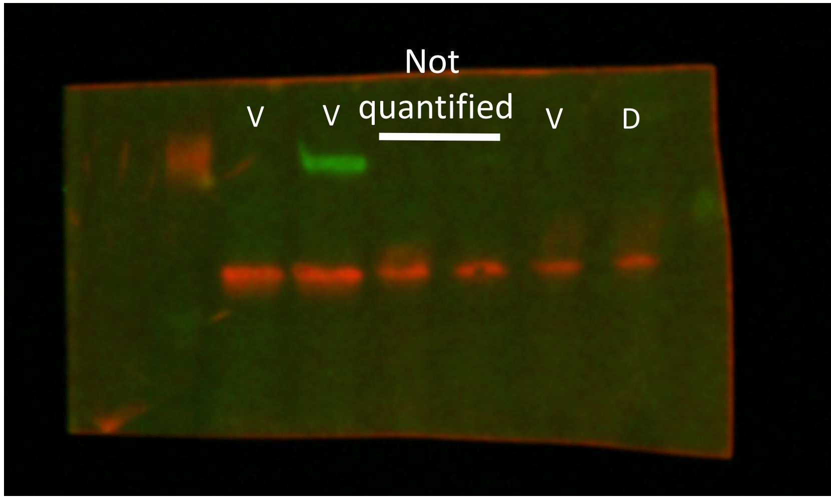
V V
Not quantified
V D

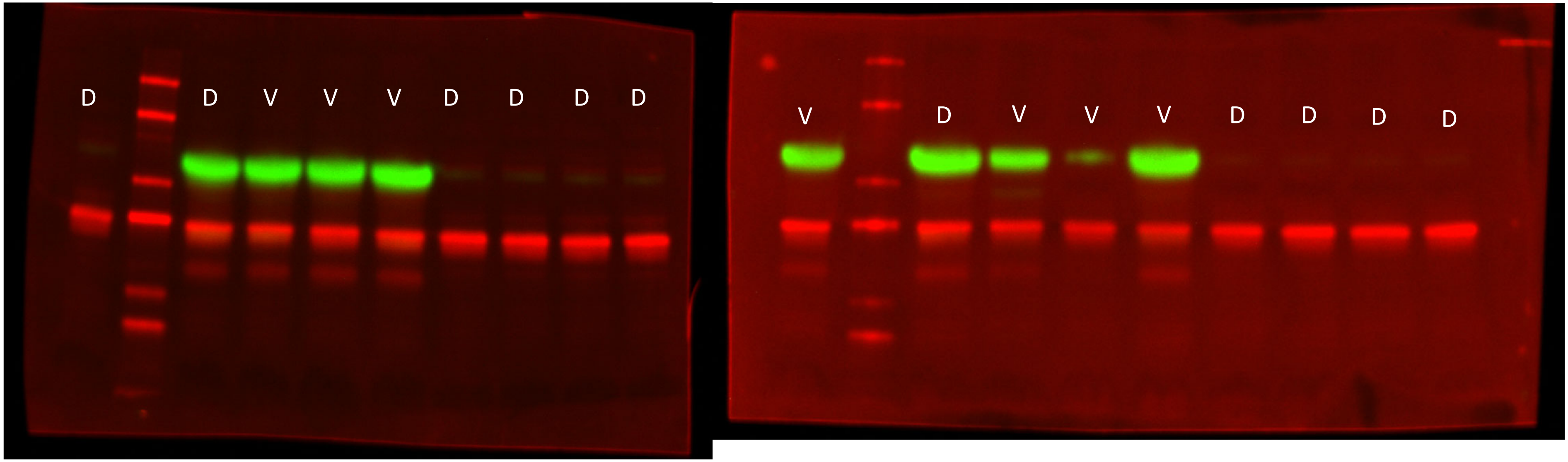

Supplemental Figure 1: Serum albumin extravasation into the cortex three days after SE was used to evaluate integrity of the blood-brain barrier. Albumin (green bands) and GAPDH (red bands) protein levels in cortical homogenates of mice treated with vehicle (V, n=20) or CCR2 antagonist (D, n=19) after kainic acid-induced status epilepticus shown over 5 blots. A total of 8 samples had running irregularities were rerun for evaluation. The 8 samples were rerun correctly and are shown in the blots.
